# Social networks and health information sharing in COVID-19 pandemic: Tetine Sentell

**DOI:** 10.1093/eurpub/ckac131.183

**Published:** 2022-10-25

**Authors:** M Phillips, R Weldon, U Patil

**Affiliations:** Office of Public Health Studies, University of Hawaii at Manoa, Honolulu, USA

## Abstract

**Background:**

Understanding health information flow in social networks is important for designing effective health communications strategies and to achieve health literacy. Limited information is known about variation in social networks and health information sharing in the COVID-19 pandemic by demographic factors. Young people are of particular interest given their heavy exposure to digital media sources, which include considerable health misinformation.

**Methods:**

Hawai'i (n = 324) residents between 18-35 completed a Spring 2021 online survey including questions on health information flow in social networks: (1) how many they talked to and (2) listened to about health. Two Poisson regression models were fit with gender, education, and race/ethnicity predicting social network size.

**Results:**

Respondents were 67.6% female. Respondents discussed their own health with 2-3 people (M = 2.18, SD = 2.95) and listened to roughly the same number. Respondents who talked with a greater number of individuals about their own health were significantly more likely to have larger networks for listening to others (r (317) = .614; p< .001). In the model for discussing their own health, as education increased so did social network size. For the model predicting discussing others’ health, gender was significant (p = 0.003); women listened to 30.6% more individuals than men. Most (73%) respondents had conducted a recent digital health search for either themselves or someone else, including for parents, grandparents, and children. Facebook (63%) and Instagram (58%) were the most popular online sources for COVID-19 health information.

**Conclusions:**

Understanding social networks and digital health sources in young people are important for designing effective health communications to reach all communities, especially those experiencing health inequities, given the amount of health misinformation circulating and the need to build trust in public health communication.

**Key messages:**

• Social networks provide access to critical health information including information obtained from digital sources.

• Gender and education were important predictors of social network size in COVID-19 health communications.

